# Vomiting as a Presenting Symptom of Infantile Vitamin B12 Deficiency

**DOI:** 10.7759/cureus.25134

**Published:** 2022-05-19

**Authors:** Sienna Wong, Nabeeha Ahmad, Allison L Rossetti

**Affiliations:** 1 Department of Internal Medicine, The Ohio State University Wexner Medical Center, Columbus, USA; 2 Department of Internal Medicine, Nationwide Children’s Hospital, Columbus, USA

**Keywords:** failure to thrive, vitamin b12 deficiency, vomiting in infants, infantile vitamin deficiency, exclusive breast feeding

## Abstract

An 8-month-old, exclusively breastfed girl presented with a five-month history of vomiting with subsequent failure to thrive and lethargy. Family history was notable for the maternal history of gastroschisis. Mother had no dietary restrictions and had successfully breastfed multiple children for >12 months without issue. Initial evaluation was notable for macrocytic anemia. Subsequent serum B_12_ levels were undetectable. Upon further questioning, the mother had significant bowel resection as an infant due to complications of gastroschisis. Maternal serum B_12 _levels were also undetectable. The infant’s symptoms resolved with supplementation.

## Introduction

Vitamin B12 is a water-soluble vitamin that plays a critical role in DNA synthesis. Deficiency in exclusively breastfed infants is well-documented and most commonly attributed to maternal deficiency related to animal product dietary restriction or malabsorption [1-3]. Deficient infants can present with various nonspecific symptoms, including failure to thrive, irritability, developmental delay, hypotonia, seizures, hyperpigmentation, and refusal of solid food [4]. Neurologic symptoms, in particular, have been well documented [2]. Here, we describe a case of vitamin B12 deficiency in an infant who initially presented with persistent vomiting and lethargy.

## Case presentation

An 8-month-old girl with a past medical history significant for ventral hernia repair at two months of age presented to the emergency department with a five-month history of non-bloody, nonbilious, non-projectile emesis. Emesis was increasing in volume and frequency since onset. Pregnancy was unremarkable, with term delivery and no history of birth trauma or cerebral palsy. The newborn screen was normal. The infant was exclusively breastfed, and the introduction of complementary foods had been attempted on several occasions without success. Mother had briefly trialed dairy elimination without improvement in symptoms but was otherwise without dietary restrictions. There was no personal or family history of autoimmune disorders.

Review of the patient’s growth chart noted a decline from the 59^th^ percentile at the age two months to the 14^th^ percentile at age seven months in weight-for-age since onset of symptoms. Additional data points were unavailable. Physical examination was notable for lethargy, hypotonia, and a soft abdomen with palpable liver edge 1cm below the costal margin. Laboratory workup was notable for mild leukopenia, macrocytic anemia with Hb 9.8 g/dL and MCV 102.3 fL, and mild elevation of aspartate aminotransferase (Table 1).

**Table 1:** Initial laboratory workup WBC: white blood cells, MCV: mean corpuscular volume, MCH: mean corpuscular hemoglobin, MCHC: mean corpuscular hemoglobin concentration, RDW: red cell distribution width, RBC: red blood cells, BUN: blood urea nitrogen, ALT: alanine aminotransferase, AST: aspartate aminotransferase

NAME		REF. RANGE	VALUE	
Routine Hematology				
	WBC	6.0 – 17.5 10*3/uL	4.7	↓
	Hemoglobin	10.5 – 13.5 g/dL	9.8	↓
	Hematocrit	33.0 – 39.0 %	26.7	↓
	Platelet count	142 – 508 10*3/uL	144	
	MCV	70.0 – 86.0 fL	102.3	↑
	MCH	23.0 -31.0 pg	37.5	↑
	MCHC	30.0 – 36.0 %	36.7	↑
	RDW	10 – 14.1 %	16.7	↑
	RBC	3.7 – 5.3 10*6/uL	2.61	↓
General Chemistry				
	Sodium	135 – 145 mmol/L	133	↓
	Potassium	3.6 – 5.9 mmol/L	4.3	
	Chloride	98 – 110 mmol/L	103	
	Carbon dioxide	21 – 30 mmol/L	22	
	BUN	5 – 18 mg/dL	6	
	Creatinine	0.15 – 0.4 mg/dL	<0.15	↓
	ALT	<40 U/L	39	
	AST	20-60 U/L	63	↑
	Total bilirubin	0.1 - 1.0 mg/dL	0.5	
	Direct bilirubin	< 0.6 mg/dL	0.2	

The patient was admitted to the general pediatrics service for further evaluation. Abdominal radiograph, abdominal ultrasound, and upper gastrointestinal series with small bowel follow-through were unremarkable. Computed tomography of the head without contrast was also unremarkable. Thyroid-stimulating hormone (TSH), anti-tissue transglutaminase antibody (anti-tTG), and serum immunoglobulin A (IgA) levels were normal. A nasogastric tube was inserted, and despite trialing both bolus and continuous feeds of breastmilk, large volume emesis persisted. Promotility drugs were not attempted. Macrocytic anemia noted in the initial investigation prompted evaluation of serum folate and B12 levels that resulted in undetectable levels of vitamin B12. Methylmalonic acid and homocysteine levels were elevated, confirming a diagnosis of vitamin B12 deficiency. Testing for inborn errors of vitamin B12 absorption and utilization were negative.

On further discussion with the patient’s mother, she had a history of gastroschisis that required resection of two-thirds of her small bowel as an infant. At the request of the general pediatrics service, the mother’s serum B12 level was also checked and found to be undetectable. Despite this, she denied any neurologic symptoms or prior workup for anemia or vitamin deficiency.

Treatment was initiated with daily intramuscular (IM) cyanocobalamin injections for seven days with resolution of vomiting and lethargy. Though vomiting resolved prior to discharge, her oral intake was inadequate for catch-up growth. She was discharged with a nasogastric tube in place to continue oral/gavage feeding regimen. She completed an additional four weeks of weekly IM cyanocobalamin injections. At outpatient follow-up three weeks following discharge, she was noted to have resumed adequate direct breastfeeding, was eating solid foods, and was demonstrating adequate weight gain without the need for gavage feeding.

## Discussion

Literature is lacking to define the incidence of vitamin B12 deficiency among infants in the United States. One study investigating deficiency among newborns secondary to maternal deficiency reports an incidence of 0.88 out of 100,000 births [5]. Symptom onset in infants of deficient mothers typically occurs between 2-12 months of age with median age of 4-5 months and a diagnostic delay of 3-4 months [1,2]. Long-term neurologic outcome depends on degree and duration of deficiency, underscoring the importance of prompt recognition, diagnosis, and treatment. As in this case, it is not uncommon for multiparous mothers to have previously exclusively breastfed children without developmental abnormalities [1]. Similarly, it is also not uncommon for mothers to be asymptomatically deficient [3]. As such, it is critical for providers to keep a high index of suspicion based on patient presentation as well as maternal medical, surgical, and social histories. Given the role of vitamin B12 in neurologic development and the potential sequelae of long-term deficiency, it may be prudent for adult providers to screen high-risk patients, even if asymptomatic, for deficiency prior to or during pregnancy.

The pathophysiology of vomiting in the setting of vitamin B12 deficiency is not well understood, although we speculate that it may be secondary to neurologic effects of deficiency on the enteric nervous system or from methylmalonic acidemia. In addition to vomiting, a refusal of complementary foods has been reported in several cases of infantile vitamin B12 deficiency with rapid resolution upon supplementation [2,3,6]. Given that these symptoms are both caused by and perpetuate deficiency, its presentation-if history provided is initially gastrointestinal with a later onset of neurologic symptoms and failure to thrive, as in this case-can mislead providers into thinking there is an underlying gastrointestinal etiology. While refusal of complementary feeding is a common phenomenon, its persistence in the presence of concomitant laboratory abnormalities and clinical symptoms can assist in recognition of B12 deficiency within this demographic. While macrocytic anemia is a late finding in the clinical course, it is more commonly seen in severe deficiency among infants than older age groups [1,7]. Honzik et al. reported the presence of elevated aminotransferases in both mild and severe deficiency, which is reflected in this case [1].

Treatment leads to rapid improvement. Supplementation with either high-dose oral, sublingual, or parenteral supplementation is effective in infants, although parenteral supplementation may be advantageous in the setting of persistent vomiting [7-9]. Empiric treatment for suspected vitamin B12 deficiency has been proposed for resource-scarce settings, particularly for young children and women of reproductive age, given the severe neurodevelopmental sequelae of prolonged and severe deficiency [10]. In resource-rich settings, clinicians should maintain a high degree of suspicion in exclusively breastfed infants even in the absence of primarily neurologic symptoms. Given the rapid clinical improvement when supplemented, it may be prudent to trial supplementation if deficiency is highly suspected even while confirmatory serum studies are pending. Though prompt initiation of treatment generally results in rapid recovery, many infants will experience long-term developmental and cognitive delays [2]. Several theories of the mechanism underlying neurologic manifestations of B12 deficiency exist, but the etiology remains poorly understood [2]. Resolution of symptoms related to deficiency typically occurs within weeks of supplementation [11].

While vitamin B12 is water-soluble and the theoretical risk of toxicity is low, there has been a case report of cutaneous and neurologic adverse effects in an adult patient when supplemented in excess, which subsequently resolved following cessation of supplementation [12]. More commonly described in various case reports of deficient infants is a transient movement disorder during the initiation of supplementation, although this was not observed in our patient. Several cases have reported variable movements without epileptiform discharges, despite potentially appearing epileptic in nature, with some even evolving over time [2,13-15]. Similar to neurologic symptoms related to deficiency, this resolves spontaneously within weeks, and its etiology remains unclear [2,14-16]. Further studies are needed to determine the optimal supplementation schedule and dosages for infantile deficiency to avoid adverse effects potentially associated with supplementation.

## Conclusions

Due to limited endogenous stores, exclusively breastfed infants are at a unique risk for vitamin B12 deficiency. Presenting symptoms may be nonspecific, and diagnosis requires a high index of suspicion. Although neurologic and hematologic complications are considered pathognomonic, they are generally late-stage findings. As outcomes are predicted by severity and duration of deficiency providers must consider the constellation of earlier clinical signs that infants may particularly present with mild deficiency. A comprehensive family medical history should be included in the assessment of an infant with failure to thrive. Importantly, prenatal and/or perinatal screening for B12 deficiency may be prudent in certain high-risk groups such as patients with longstanding vegetarian or vegan diets, prior gastrointestinal surgery or pathology such as celiac disease or inflammatory bowel disease, prolonged metformin or proton-pump inhibitor use, or those with a personal or family history of pernicious anemia.
